# aThe characteristics of glucose metabolism in the sulfonylurea receptor 1 knockout rat model

**DOI:** 10.1186/s10020-018-0067-9

**Published:** 2019-01-07

**Authors:** Xiaojun Zhou, Chunmei Xu, Zhiwei Zou, Xue Shen, Tianyue Xie, Rui Zhang, Lin Liao, Jianjun Dong

**Affiliations:** 1 Department of Endocrinology, Shandong Provincial Qianfoshan Hospital, Shandong University, Jinan, Shandong 250014 People’s Republic of China; 2 Department of Endocrinology, Qilu Hospital of Shandong University, Shandong University, Jinan, Shandong 250012 People’s Republic of China; 3 Department of Endocrinology, Shandong Provincial Qianfoshan Hospital, Shandong University of Traditional Chinese Medicine, Jinan, Shandong China

**Keywords:** Sulfonylurea receptor 1, Knockout rat model, Glucose metabolism, Insulin sensitivity

## Abstract

**Background:**

Sulfonylurea receptor 1 (SUR1) is primarily responsible for glucose regulation in normal conditions. Here, we sought to investigate the glucose metabolism characteristics of *SUR1*^*−/−*^ rats.

**Methods:**

The TALEN technique was used to construct a *SUR1* gene deficiency rat model. Rats were grouped by *SUR1* gene knockout or not and sex difference. Body weight; glucose metabolism indicators, including IPGTT, IPITT, glycogen contents and so on; and other molecule changes were examined.

**Results:**

Insulin secretion was significantly inhibited by knocking out the *SUR1* gene*. SUR1*^−/−^ rats showed lower body weights compared to wild-type rats, and even *SUR1*^*−/−*^ males weighed less than wild-type females. Upon *SUR1* gene knockout, the rats showed a peculiar plasma glucose profile. During IPGTT, plasma glucose levels were significantly elevated in *SUR1*^*−/−*^ rats at 15 min, which could be explained by SUR1 mainly working in the first phase of insulin secretion. Moreover, *SUR1*^*−/−*^ male rats showed obviously impaired glucose tolerance than before and a better insulin sensitivity in the 12th week compared with females, which might be related with excess androgen secretion in adulthood. Increased glycogen content and GLUT4 expression and the inactivation of GSK3 were also observed in *SUR1*^*−/−*^ rats, which suggested an enhancement of insulin sensitivity.

**Conclusions:**

These results reconfirm the role of SUR1 in systemic glucose metabolism. More importantly, our *SUR1*^*−/−*^ rat model might be applied in other fields, such as for exploring other hypoglycaemic functions of sulfonylureas.

**Electronic supplementary material:**

The online version of this article (10.1186/s10020-018-0067-9) contains supplementary material, which is available to authorized users.

## Background

The sulfonylurea receptor (SUR) belongs to the ATP-binding cassette (ABC) family of proteins and appears in several isoforms: SUR1 in pancreatic β-cells and neurons, SUR2A in cardiac and skeletal muscle, and SUR2B in vascular smooth muscle (Seino & Miki, 2003). SUR1 is unique and serves as an ion channel regulator, in which ATP hydrolysis modulates the gating of a separate Kir6.2 channel pore (Aguilar-Bryan et al., 1995; Aittoniemi et al., 2009). SUR1, together with pore-forming Kir6.2, comprises the ATP-sensitive K^+^ (KATP) channel, which serves to transduce metabolic changes into biophysical signals through the following sequence of events (Aguilar-Bryan et al., 1995): elevated glucose increases the cytoplasmic [ATP]: [ADP] ratio in pancreatic β-cells, which causes closure of the KATP channels, β-cell membrane depolarization, opening of the voltage-dependent Ca^2+^ channels and Ca^2+^ influx. The resultant rise in the intracellular Ca^2+^ concentration triggers exocytosis of insulin granules and insulin secretion (Remedi et al., 2006).

Furthermore, SUR1 plays a key role in cellular and microvascular dysfunction in various forms of central nervous system (CNS) injury (Simard et al., 2010). In situations of mechanical stress, inflammation, hypoxia, an upregulated SUR1 expression could be observed (Simard et al., 2012). A recent study revealed that post-injury SUR1 upregulation in the hippocampus was associated with a later disturbance in learning (Patel et al., 2010). Hence, it is clear that research on the specific functions of SUR1 is valuable.

Thanks to various animal models, we have gained a better understanding of the aetiology, pathology, and molecular mechanisms of different disorders (Blesa et al., 2012). Over the past few decades, the adoption of molecular biology techniques for the genetic manipulation of rodents has resulted in a surge of interest in using these rodents as model systems for the investigation of almost all facets of mammalian biology (Sauer, 1998). Not only does this allow the direct assessment of gene function in intact animals, it also allows the design of increasingly useful animal models of human disease (Sauer, 1998). Therefore, we can apply gene knockout technology to explore the special function of the *SUR1* gene in mammalian biology. Currently, there have been studies of *SUR1* knockout mice in a variety of pathological conditions, such as persistent hyperinsulinaemic hypoglycaemia of infancy (PHHI) (Miki et al., 1999) and a severe dysregulation of glucose homeostasis known as congenital hyperinsulinism (CHI) (Straub et al., 2001; Dunne et al., 2004; Bryan et al., 2007; Nakamura & Bryan, 2014). Although much effort has been given in this field by using the *SUR1* gene knockout mice, no direct evidence has been available for rats. When compared with mice, rats are closer to humans in cognitive behaviour and often serve as an ideal animal model of hypertension (Rimbaud et al., 2011; Fennell et al., 2002), diabetes mellitus (Reed et al., 2000; Chis et al., 2009), breast carcinoma (Mendes et al., 2005) and neurological disorders (McGonigle, 2014). In addition, considering that such studies require invasive procedures and large blood and tissue samples, the large body size of rats could be an advantage over the smaller mice (Srinivasan & Ramarao, 2007).

Therefore, we established a *SUR1*^*−/−*^ rat model via the TALEN technique and investigated changes in glucose metabolism when knocking out the *SUR1* gene, which was primarily responsible for glucose regulation under normal conditions. The results clearly demonstrated that *SUR1*^*−/−*^ rats showed a peculiar glucose profile. Additionally, the body weights of the model rats at four weeks of age were less than that of wild-type animals, and they remained significantly smaller than the wild-type throughout adulthood. Intriguingly, *SUR1*^*−/−*^ male rats weighed even less than wild-type female rats. More importantly, we obtained novel insights into the metabolism of glucose that chiefly manifested as enhanced insulin sensitivity. Due to the differences in hormones, *SUR1*^*−/−*^ male and female rats exhibited different glucose metabolism characteristics.

## Methods

### Animal model for *SUR1* gene knockout

The *SUR1* gene ID was searched in the PubMed genomic database and was found as 25,559. The *SUR1* null Sprague-Dawley rat lines were acquired by TALEN-mediated gene targeting from Cyagen Biosciences Inc., Guangzhou, China. The detailed procedure included the designation and building of the TALEN vector, transcription to mRNA, pronucleus microinjection of mRNA and acquisition of the chimaera rat model.

Rats of the 5th generation or greater were used in the experiments. Genotypes were determined by Sanger sequencing of PCR products for each rat to confirm deletion sites. The SUR1 protein expression was examined by western blotting to further corroborate the successful deletion of the *SUR1* gene. The animals were housed at 22 °C with 50% humidity and a 12 h light-dark cycle. All rats were free-fed with standard diet (20% crude protein, 3% crude fibre, 3% crude fat, remaining 74% carbohydrate and microelement; Beijing Keaoxieli Feed Co., Ltd., China), and the drinking water was changed four times per week.

### Experimental groups

All experimental rats in the study were divided into four groups as follows: *SUR1*^*−/−*^ male rats, *SUR1*^*−/−*^ female rats, wild-type male rats and wild-type female rats. The weight of the rats ranged from 60 to 140 g, with an age of four weeks at the beginning of the experiments. After 8 weeks of feeding with the standard diet, all rats were euthanized, and the organs were collected for multiple biochemical analyses. All experimental procedures were performed in accordance with the animal protocols approved by the ethics committee of Qianfoshan Hospital Affiliated to Shandong University.

### Immunofluorescent double staining for insulin and glucagon

Pancreas tissues from the four groups were extracted for immunofluorescence staining to evaluate the expression of insulin and glucagon. The free-floating sample sections were incubated with anti-insulin antibody (1:100, #8138, Cell Signaling Technology) and anti-glucagon antibody (1:100, 15,954–1-AP, Proteintech). Immunoreactivity products were visualized by incubation with appropriate Alexa Fluor 488-conjugated secondary antibodies (1:50; Invitrogen, CA, USA), 594-conjugated secondary antibodies (1:150; Invitrogen), along with DAPI stain to visualize the nuclei. All steps were performed using 0.3% Triton X-100 in phosphate-buffered saline (PBS). After staining, images were taken at randomly selected fields using an OLYMPUS FSX100 imaging system (Olympus, Tokyo, Japan).

### Body weight

After initiation of experiments with 4-week-old rats, the rats were weighed once per week until the age of 12 weeks.

### Fasting plasma glucose (FPG)

All experimental rats of the four groups were fasted for 12 h with access to drinking water. The next day, the FPG level of each rat was detected. Blood samples were collected sequentially from the tail vein, and plasma glucose was measured with a One-Touch Glucometer (Ascensia Breeze, Bayer, Germany). FPG was determined once per week for 8 weeks.

### IPGTT and IPITT

An intraperitoneal glucose tolerance test (IPGTT) was performed to evaluate glucose tolerance after the rats were fasted for 12 h. The following day, rats were injected with a bolus of glucose (1 g/kg i.p.), and blood from the tail vein was tested at 0, 15, 30, 60, and 120 min from injection by a One-Touch Glucometer (Ascensia Breeze, Bayer, Germany).

To evaluate the insulin tolerance of the rats, an intraperitoneal insulin tolerance test (IPITT) was carried out after they were fasted for 4 h. A bolus of insulin (1 unit/kg i.p.) was administered to the animals, and blood samples were collected as described above. The mean area under the curve (AUC) was calculated as the primary result of the insulin tolerance test. IPGTT and IPITT experiments were performed every four weeks.

### PAS staining for glycogen analysis

After the livers were removed, tissue sections with a thickness of 4 μm were prepared and fixed in 10% neutral buffered formalin, followed by embedding in paraffin. The glycogen content was determined by periodic acid-Schiff (PAS) staining. After hydration, the paraffin sections were incubated in periodic acid for 5 min, followed by washing, and were then stained with Schiff’s reagent for 15 min, followed by washing, and were finally dyed with haematoxylin. Sections from three animals per group and five sections per treatment were analysed. The muscle glycogen content was also detected as described above.

### Western blotting analyses

After euthanasia, tissues including liver and muscle were removed and homogenized with RIPA using a homogenizer, and the tissue proteins were isolated. Western blotting was performed with antibodies against glucose transporter 4 (GLUT4) (1:1000, ab65267, Abcam) for muscle tissue. The level of glycogen synthase kinase 3 (GSK3) (1:1000, #5080, Cell Signaling Technology) and phosphorylated glycogen synthase kinase 3 (PGSK3) (1:1000, #9331, Cell Signaling Technology) were detected for liver tissue. β-actin (1:5000, 60,008–1-Ig, Proteintech) was used as a reference protein.

### Immunofluorescent staining for GLUT4

Muscle sections were extracted for immunofluorescence staining to evaluate the expression of GLUT4. The free-floating sample sections were incubated with anti-GLUT4 antibody (1:500, ab65267, Abcam) overnight. The sections were then washed 3 times for 5 min in PBS. Secondary antibodies were applied to the sections for 1 h at room temperature. GLUT4 antibody was targeted with Alexa Fluor 594-conjugated secondary antibodies (1:200; Invitrogen). DAPI staining for cell nuclei was added to the secondary antibody solution. All steps were performed using 0.3% Triton X-100 in PBS. After staining, images were taken at randomly selected fields using an OLYMPUS FSX100 imaging system.

### Image quantification analysis

Quantification of immunofluorescence image was performed by converting images to grayscale, inverting their colour and quantifying field staining intensity with Image-Pro Plus 6.0 software (Media Cybernetics, Inc., Bethesda, MD, USA). For quantification, three randomly selected high-power fields (HPFs) (× 400 for immunofluorescence studies) were analysed in each section. The mean red fluorescence (glucagon) and green fluorescence (insulin) intensity per HPF for each rat were then determined. To quantify the immunoreactivity of proteins of interest, for each treatment 10–15 photographs were taken at × 400 magnification. The same approach applied to the analysis of immunofluorescence staining for GLUT4 in muscle.

The quantification of the glycogen content in liver and muscle was also performed by using Image-Pro Plus 6.0 software. Semiquantitative analysis was performed by visual analysis of PAS-stained cross-sectional area tissue sections from three randomly selected fields. Altogether, five sections from each animal were analysed. Thus, fifteen images were analysed in each animal. The mean optical density was scaled by the integrated optical density (IOD) divided by area of selected region to determine an average glycogen content in these regions. The captured images were coded and quantified in a blinded manner.

### Data and statistical analysis

Statistical analysis was performed using SPSS Statistics 22.0 (SPSS Inc., Chicago, USA). Results are expressed as means ± standard error of mean (SEM) and the level of statistical significance was estimated at *P* < 0.05.

The data of immunofluorescence staining, and hepatic and muscle glycogen content were quantified by Image-Pro Plus 6.0 software. And two-sample t-test for independent groups was used to identify any significant differences.

Data of PGSK3/GSK3 level and GLUT4 level by western blotting were normalized by taking values obtained for wild-type females as 1 and by calculating a corresponding proportional value for wild-type males and both sexes of *SUR1*^*−/−*^ rats. Normalized data were analysed by two-sample t-test for independent groups.

In addition, the charts were prepared using GraphPad Prism 5.0 (GraphPad Software, Inc., La Jolla, CA). Differences between two groups were also assessed performed with two-sample two-tailed t test for body weight, body weight gain, FPG, body glucose in the 4th, 8th and 12th week of IPGTT and AUC of body glucose in the 4th, 8th and 12th week of IPITT, respectively.

## Results

### Identification of *SUR1* gene knockout

**Fig. 1:**
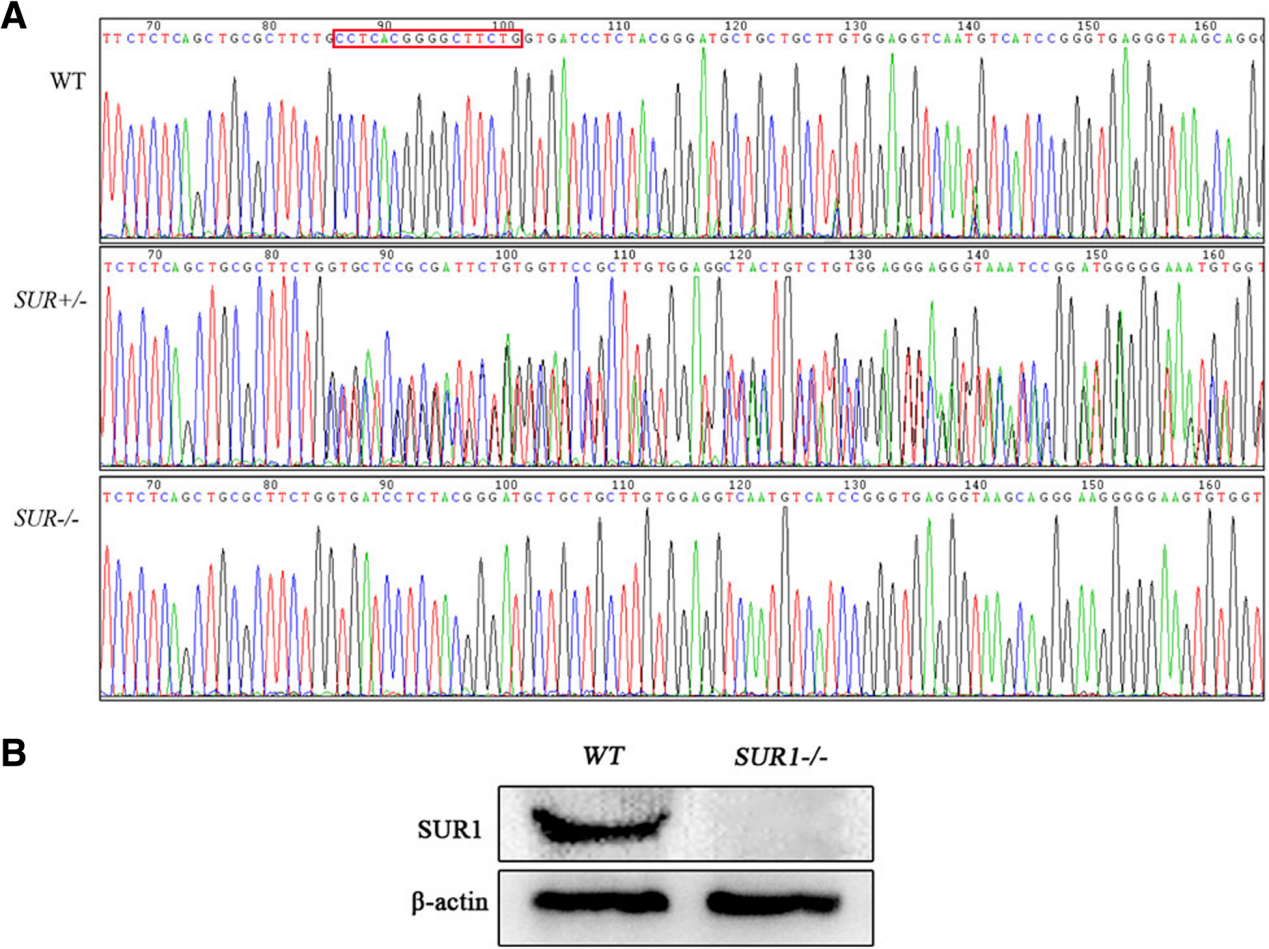
Sanger gene sequencing and SUR1 protein expression in *SUR1*^*−/−*^ and wild-type rats. **a** Sanger gene sequencing results showed that *SUR1*^*−/−*^ rats have a partial base deletion in the region of the *SUR1* gene. **b** Western blotting revealed that no expression of SUR1 protein was observed in *SUR1*^*−/−*^ rats

Sanger sequencing of the PCR products from each rat showed a 16 bp deletion of the *SUR1* gene in the *SUR1*^*−/−*^ rats corresponding to CCT CAC GGG GCT TCTG compared with wild-type rats. This indicated that knocking out the *SUR1* gene homozygote was successful (Fig. 1a). Additionally, as shown in Fig. 1b, *SUR1* knockout rats had no SUR1 protein expression compared with wild-type rats, further confirming that the *SUR1* gene knockout model was successfully constructed. The final TALEN vector for deletion of the *SUR1* gene is presented in Additional file 1.

### Animals

Six rats of every group were included in this study, and there were no accidental deaths over the duration of the experiment.

### An obviously reduced insulin expression in the pancreas was observed in *SUR1*^*−/−*^ rats

**Fig. 2:**
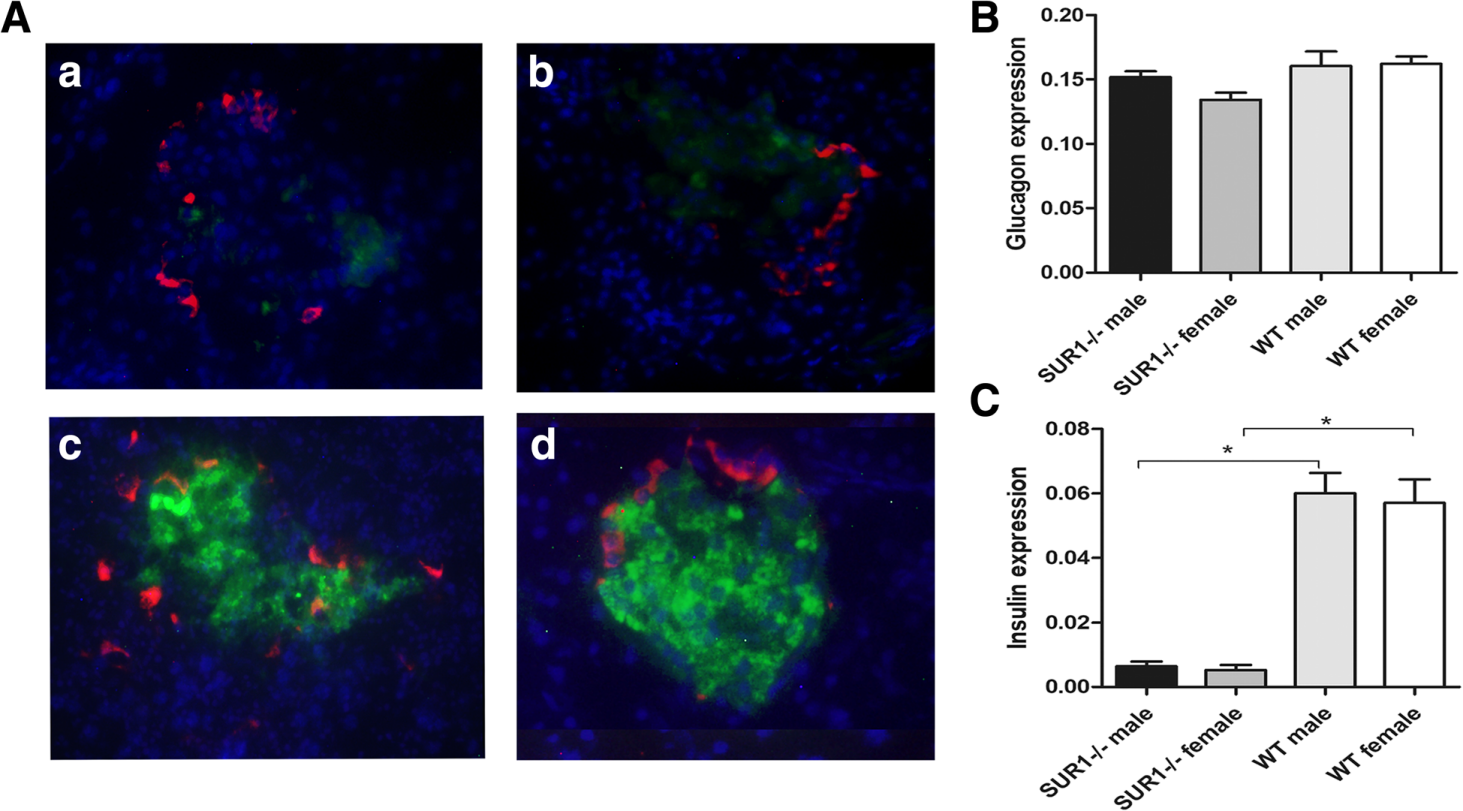
Expression and colocalization of insulin and glucagon in *SUR1*^*−/−*^ and wild-type rats. Immunofluorescent colocalization of insulin and glucagon in *SUR1*^*−/−*^ male rats (a), *SUR1*^*−/−*^ female rats (b), wild-type male rats (c) and wild-type female rats (d). Pancreatic sections were immunostained with anti-insulin antibody (green) and anti-glucagon antibody (red). *SUR1*^*−/−*^ rats showed obviously reduced insulin expression with unchanged glucagon expression compared to wild-type rats. (**A**) Immunofluorescent staining for glucagon and insulin. (**B**) Quantitative analysis of glucagon expression. (**C**) Quantitative analysis of insulin expression. The values denote the means ± SEM. *: *P* < 0.05. Original magnification, × 400

The effect of *SUR1* gene knockout on insulin and glucagon secretion was further examined by immunofluorescence, where a reduced fluorescence staining for insulin was observed in both *SUR1*^*−/−*^ male rats and *SUR1*^*−/−*^ female rats, without changes of glucagon expression, compared with wild-type rats (Fig. 2). Reduced fluorescent staining for insulin further confirmed that the *SUR1* gene knockout induced decreased insulin secretion.

### *SUR1*^*−/−*^ rats showed lower body weights

**Fig. 3:**
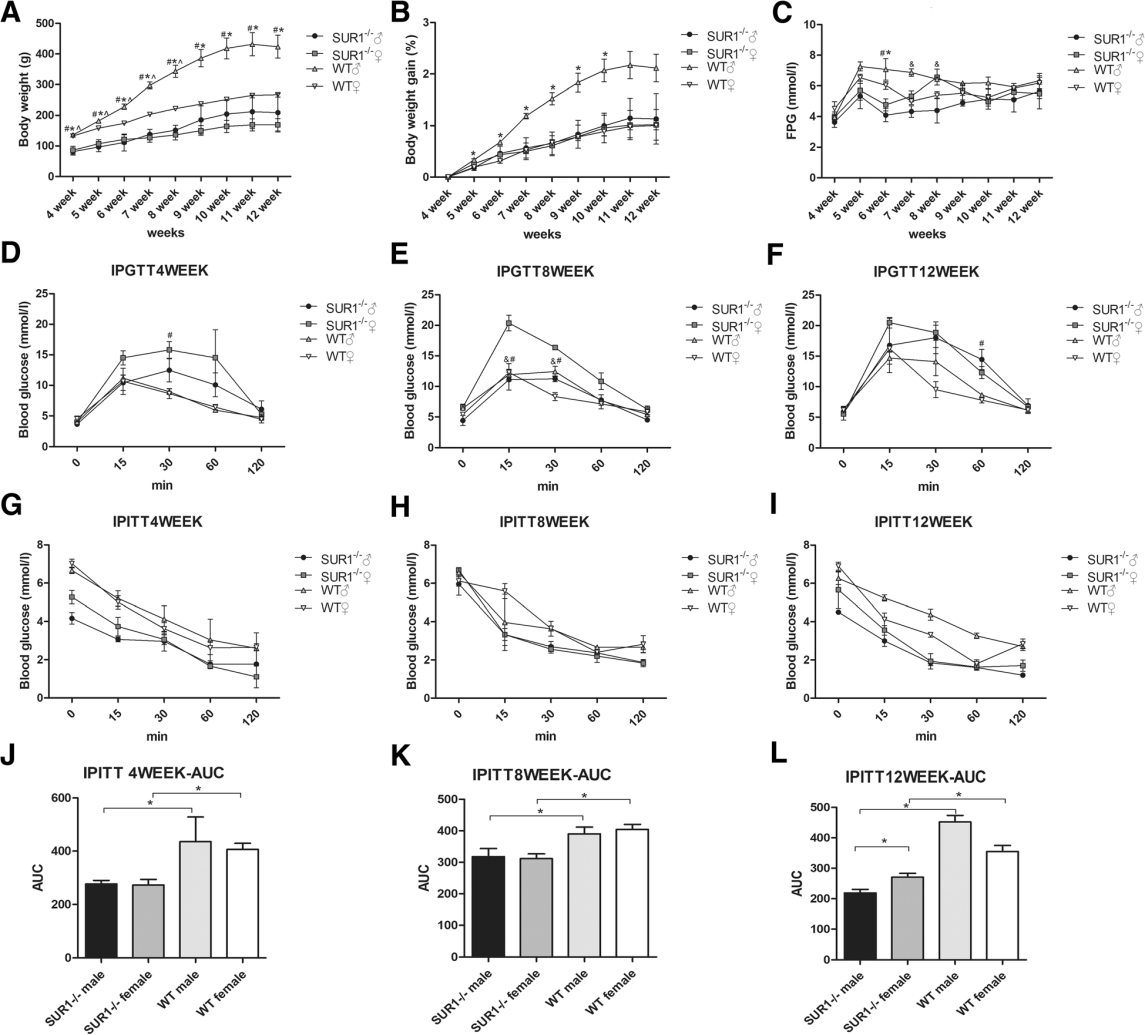
Body weight, body weight gain, FPG, IPGTT, IPITT and AUC during IPITT.**a** Body weights of *SUR1*^*−/−*^ rats at the beginning of the experiment were 35–40% (male 40%, female 35%) less than those of the wild-type group, and they remained significantly smaller than the wild-type throughout adulthood. **b** The curve showed that the body weight of *SUR1*^*−/−*^ male rats gained significantly slower than wild-type male rats. **c** *SUR1*^*−/−*^ rats were significantly more hypoglycaemic than wild-type rats when fasted for 12 h. Among the *SUR1*^*−/−*^ rats, the male showed lower fasting glucose levels than those of females at the age of 7 and 8 weeks. **d-f** *SUR1*^*−/−*^ rats displayed impaired glucose tolerance, and blood glucose levels of the *SUR1*^*−/−*^ rats were significantly higher than those of the wild-type at 15 min. Compared to *SUR1*^*−/−*^ males, female rats showed a more severely impaired glucose tolerance curve. **g-l** *SUR1*^*−/−*^ rats were insulin-hypersensitive, and a rapid and sustained reduction in serum glucose was observed in *SUR1*^*−/−*^ rats after injecting insulin. The values denote the means ± SEM. &: *P* < 0.05, *SUR1*^*−/−*^ male versus *SUR1*^*−/−*^ female. #: *P* < 0.05, *SUR1*^*−/−*^ female versus wild-type female. *: *P* < 0.05, *SUR1*^*−/−*^ male versus wild-type male. ^: *P* < 0.05, *SUR1*^*−/−*^ male versus wild-type female

The body weights of *SUR1*^*−/−*^ rats at the age of 4 weeks were 35–40% (male 40%, female 35%) less than that of the wild-type group, and they remained significantly smaller than the wild-type rats throughout adulthood (Fig. 3a). Compared with wild-type male rats, *SUR1*^*−/−*^ male rats weighed less (*P* = 0.05), and a similar result was obtained between the *SUR1*^*−/−*^ and wild-type female rats (*P* < 0.01). Intriguingly, a significantly lower body weight (*P* < 0.01) was observed for *SUR1*^*−/−*^ male rats compared to wild-type female rats. Body weight gain curves showed that the body weight of *SUR1*^*−/−*^ male rats gained significantly slower than that of wild-type male rats (*P* < 0.05) (Fig. 3b).

### *SUR1*^*−/−*^ rats were glucose intolerant and exhibited enhanced insulin sensitivity

As shown in Fig. 3c, *SUR1*^*−/−*^ rats were significantly more hypoglycaemic than wild-type rats when fasted for 12 h (4.10 ± 0.75 vs 7.07 ± 1.22 mM for male, *P* < 0.05; 4.77 ± 0.67 vs 6.03 ± 0.49 mM for female, *P* < 0.05) at the age of 6 weeks. In addition, among the *SUR1*^*−/−*^ rats*,* the males showed a lower FPG than the females (4.40 ± 1.41 vs. 6.57 ± 0.91 mM, *P* < 0.05) at the age of 7 and 8 weeks.

IPGTT was performed at the 4th, 8th, and 12th week to measure the glucose tolerance of the experimental rats (Fig. 3d**-**f). Plasma glucose levels were significantly elevated in *SUR1*^*−/−*^ rats at 15 min, and peak glucose values were significantly higher for *SUR1*^*−/−*^ female rats than wild-type females (20.37 ± 2.25 vs 12.28 ± 2.84 mM, *P* = 0.01) (Fig. 3e). The glucose clearance time was longer than that of the wild-type rats, which had identical baseline glucose levels and a relatively normal response to the glucose load. In addition, *SUR1*^*−/−*^ rats failed to release insulin in response to the glucose challenge compared with wild-type rats (Additional file 2).

The IPGTT results showed that *SUR1*^*−/−*^ female rats had a more severely impaired glucose tolerance compared to *SUR1*^*−/−*^ males (20.37 ± 2.25 mM vs 11.10 ± 2.92 mM, *P* < 0.05) (Fig. 3e). The IPGTT curve for *SUR1*^*−/−*^ females is steeper from 0 to 15 min at the age of 4 and 8 weeks than that of *SUR1*^*−/−*^ males (Fig. 3d and e). Although glucose levels increased faster in *SUR1*^*−/−*^ males at the age of 12 weeks, they were still not as high as that of *SUR1*^*−/−*^ females (Fig. 3f). As the time increased, the results tended to coincidence with the findings seen at the beginning of the experiment, and they indicated a significantly impaired glucose tolerance of *SUR1*^*−/−*^ rats, especially for female rats.

Following intraperitoneal injection of insulin, wild-type rats showed a typical decrease and rebound in serum glucose after insulin challenge, whereas *SUR1*^*−/−*^ rats showed a rapid and sustained reduction in serum glucose (Fig. 3g**-**i). The decrease of AUC during IPITT represents the improvement of insulin tolerance. *SUR1*^*−/−*^ rats exhibited a stronger reduction of the AUC for blood glucose levels in response to insulin compared with wild-type rats (*P* < 0.01 both for male and female) which demonstrated a significantly enhanced insulin sensitivity (Fig. 3j**-**l). In addition, a better insulin sensitivity was observed in *SUR1*^*−/−*^ males than in *SUR1*^*−/−*^ females (*P* < 0.05) (Fig. 3l).

### Glycogen levels were markedly increased and hepatic GSK3 inactivation was observed in the *SUR1*^*−/−*^ rats

**Fig. 4:**
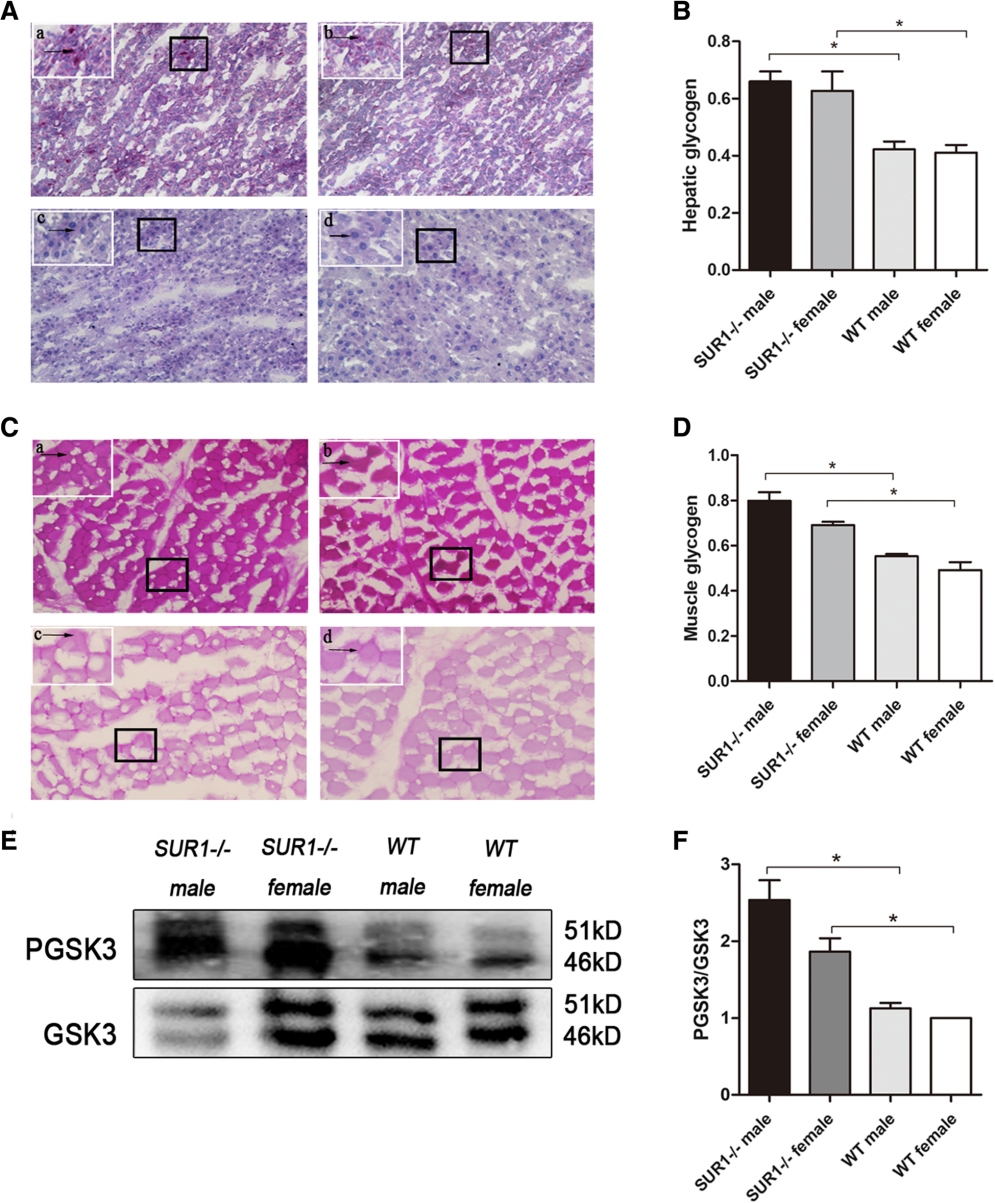
Hepatic and muscle glycogen content and alteration of hepatic GSK3 in *SUR1*^*−/−*^ and wild-type rats. *SUR1*^*−/−*^ rats showed increased hepatic (**A** and **B**) and muscle (**C** and **D**) glycogen which were stained as purple particles and arrow heads were pointing at, and the PGSK3/GSK3 ratio (**E** and **F**) in the liver was significantly increased in *SUR1*^*−/−*^ rats. a: *SUR1*^*−/−*^ male; b: *SUR1*^*−/−*^ female; c: wild-type male; d: wild-type female. The values denote the means ± SEM. *: *P* < 0.05. Images of PAS staining for glycogen were taken at × 200 magnification; a small part was magnified to × 400 and is shown on the top left corner of each picture

Glycogens were identified by PAS staining, which stains stored glycogen as purple particles. As shown in Fig. 4A**-**D, the levels of hepatic glycogen and muscle glycogen were significantly higher in the *SUR1*^*−/−*^ rats than in the wild-type rats. Furthermore, the expression and activity of GSK3, a key target for hepatic insulin sensitivity, were examined. The results suggested a significant reduction in GSK3 expression, whereas the PGSK3/GSK3 ratio was significantly increased in the *SUR1*^*−/−*^ rats (*P* < 0.05), which indirectly reflected the improvement of insulin signalling and insulin sensitivity (Fig. 4E and F).

### Expression of GLUT4 in muscle tissue was significantly enhanced in *SUR1*^*−/−*^ rats

**Fig. 5:**
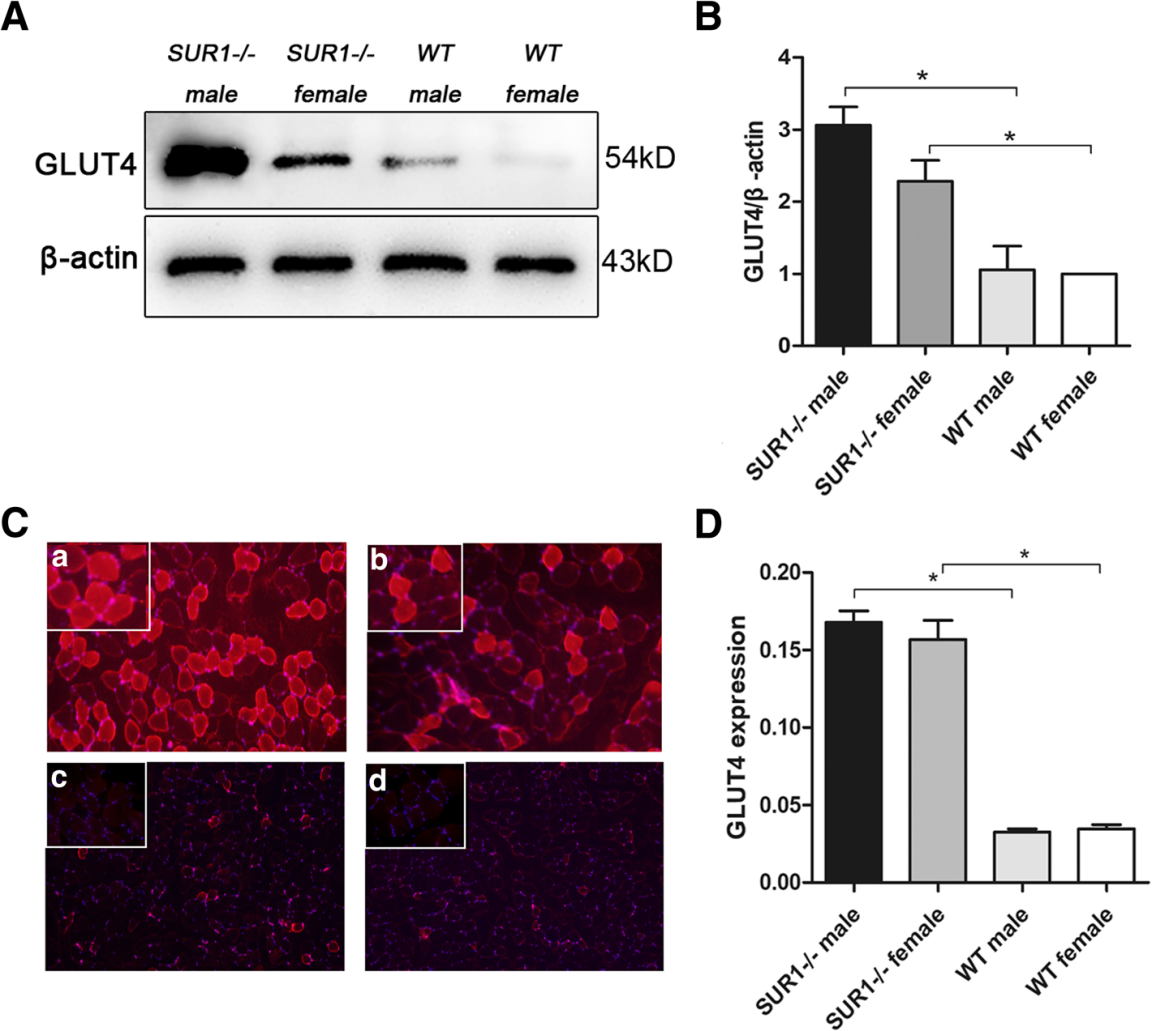
GLUT4 expression level in the muscle of *SUR1*^*−/−*^ and wild-type rats. Western blotting (**A** and **B**) and immunofluorescence (**C** and **D**) revealed that GLUT4 expression in the muscle was markedly increased in *SUR1*^*−/−*^ rats when compared with wild-type rats. a: *SUR1*^*−/−*^ male; b: *SUR1*^*−/−*^ female; c: wild-type male; d: wild-type female. The values denote the means ± SEM. *: *P* < 0.05. Images of immunofluorescence were taken at × 200 magnification; a small part was magnified to × 400 and is shown on the top left corner of each picture

Glucose uptake in the peripheral tissues is primarily mediated by GLUT4, which is a crucial pathway to mediate glucose transport. To further clarify the insulin sensitivity in the peripheral tissues of the experimental animals, the level of GLUT4 expression in muscle tissue was examined for both the *SUR1*^*−/−*^ and wild-type rats. As shown in Fig. 5A and B, GLUT4 expression in the muscle of *SUR1*^*−/−*^ rats was significantly increased by 2-fold (*P* < 0.01) compared to that of the wild-type group. The difference of GLUT4 expression between *SUR1*^*−/−*^ rats and wild-type rats was further confirmed by immunofluorescence. In accordance with the above western blotting results, increased fluorescence staining was observed in *SUR1*^*−/−*^ rat muscle (Fig. 5C and D).

## Discussion

In this study, a unique *SUR1*^*−/−*^ rat model was constructed and glucose metabolism characteristics were elucidated. Our *SUR1*^*−/−*^ rats showed a lower body weight and a peculiar plasma glucose profile, being significantly more hypoglycaemic when fasted, and obviously more hyperglycaemic when glucose-loaded compared with wild-type animals. Moreover, improved insulin sensitivity was observed, accompanied by an enhancement of hepatic and muscle glycogen levels, GSK3 inactivation and increased GLUT4 expression levels. Due to differences in hormones, *SUR1*^*−/−*^ male and female rats did not exhibit the exactly same glucose metabolism characteristics. *SUR1*^*−/−*^ male rats showed better insulin sensitivity in the 12th week compared to females, possibly due to excess androgen secretion in adulthood.

In our study, genetic ablation of the *SUR1* gene resulted in impaired glucose-stimulated insulin secretion and lower body weights of rats. Under physiological conditions, organisms use insulin-like signalling systems to control cell proliferation (Pollak, 2012), and insulin is known to have an effect on body weight promotion (Griffen et al., 1987), whereas *SUR1* gene knockout impaired the insulin secretion and contributed to the growth retardation of *SUR1*^*−/−*^ rats.

*SUR1*^*−/−*^ rats were more hypoglycaemic when fasted overnight, which could be explained by their inability to rapidly repolarize their β-cells and reduce the excess insulin release (Nenquin et al., 2004), as do *SUR1*^*−/−*^ mice (Seghers et al., 2000). Furthermore, the genetic deletion of *SUR1* caused loss-of-function mutations of the KATP channel (Shimomura et al., 2013; Hussain, 2005) and led to permanent membrane depolarization and continuous, unregulated basal insulin release (Shimomura et al., 2013), which may ultimately result in the fasting hypoglycaemia of *SUR1*^*−/−*^ rats. However, no increased basal insulin secretion, but a higher HOMA-β level was found in *SUR1*^*−/−*^ rats (Additional file 3), which indicated an enhanced β-cell function under non-stimulated conditions possibly accounting for lower fasting glucose levels of *SUR1*^*−/−*^ rats.

Regarding glucose control, *SUR1*^*−/−*^ rats showed impaired glucose tolerance, which indicated that there is little insulin release after the intraperitoneal injection of glucose into the *SUR1*^*−/−*^ animals (Seghers et al., 2000; Nakazaki et al., 2002). A sustained high level of glucose and longer clearance times after intraperitoneal injection were observed in the model animals, which indicates a delayed insulin response to glucose stimulation (Seghers et al., 2000). Additionally, intraperitoneal administration of glucose to the *SUR1*^*−/−*^ rats resulted in a marked intolerance of glucose at 15 min, which was represented as damage of the first phase of glucose-stimulated insulin secretion (Moynihan et al., 2005). In general, under hyperglycaemic conditions, plasma insulin secretion is biphasic with an early burst of insulin release during the first 10 min after a sudden rise in plasma glucose (Fehse et al., 2005), which was a key feature of first-phase insulin secretion, followed by a gradually progressive increase in plasma insulin concentration, which is the main process of second-phase insulin secretion (DeFronzo et al., 1979). The first phase of glucose-induced insulin secretion is mainly mediated by a triggering pathway (Henquin, 2009), which acts as a cornerstone for insulin secretion and involves a sequence of events including changes of metabolism, closure of KATP channels, depolarization, influx of Ca^2+^ and a rise in cytosolic free Ca^2+^ concentration (Henquin, 2009). Thus, we speculate that the SUR1 subunit of KATP channels plays a key role in the first phase of glucose-induced insulin secretion.

Following intraperitoneal injection of insulin, the *SUR1*^*−/−*^ rats showed a rapid and sustained reduction in serum glucose compared with wild-type rats, which indicated a compensatory enhancement of insulin sensitivity despite a reduction of sufficient insulin secretion and was different from no increased insulin sensitivity of *SUR1*^*−/−*^ mice (Seghers et al., 2000). Our explanation for the species differences is that *SUR1* gene may act as a more important part in rats than mice. The glucose-lowering effect of insulin was increased in *SUR1*^*−/−*^ rats and could protect *SUR1*^*−/−*^ rats from developing hyperglycaemia under glucose loading; this was in accordance with the previous study by Miki T et al. (Miki et al., 1998).

Intriguingly, compared to females, the male *SUR1*^*−/−*^ rats manifested better insulin sensitivity. This evidence corroborates the hypothesis that male androgen levels could improve insulin resistance by reducing inflammatory cytokines, such as tumour necrosis factor-α (TNF-α), that are produced by adipocytes (Wong et al., 2015). Androgen levels gradually decreased with increasing body weight (Wong et al., 2015) and insulin levels (Ahn et al., 2013). Regarding the data from this study, we speculate that a decrease in insulin secretion and a lower body weight due to knocking out the *SUR1* gene induced an elevated testosterone level, which could then reduce inflammatory cytokines and further improve insulin sensitivity (Pittas et al., 2004).

Under normal physiological conditions, plasma glucose remains within a normal range in individuals regardless of fasting or feeding. This exquisite control is governed by several integral processes, including glucose absorption, glycogen production by the liver and uptake and metabolism by peripheral tissues (Saltiel & Kahn, 2001). Glucose stimulation increased insulin concentrations due to closure of the KATP channels, which signalled the liver to regulate glucose metabolism mainly by suppressing hepatic glucose production (HGP) in response to a meal (Sharabi et al., 2015). When insulin resistance occurs, insulin is incapable of regulating HGP in the liver and fails to phosphorylate GSK3, which can be a surrogate marker of the development of an insulin-resistant state (Lee & Kim, 2007). In our study, *SUR1*^*−/*−^ rats showed an increased storage of hepatic glycogen, and this increase was accompanied by an inactivation of GSK3, thereby instituting the hepatic insulin sensitivity-improving effect.

Apart from inhibiting HGP, insulin serves as the primary regulator of blood glucose concentrations by regulating glucose uptake in muscle tissue (Saltiel & Kahn, 2001). Nearly 90% of the insulin-mediated glucose is taken up by skeletal muscle (Hinkley et al., 2014). Glucose uptake is mediated by GLUTs in most cells and GLUT4 is the predominant transporter in the muscles (Bogan, 2012). Our *SUR1*^*−/−*^ rats exhibited increased expression levels of GLUT4 in muscle tissue. Miki T et al. proposed that apart from promoting insulin secretion, KATP channel was involved in glucose transport in skeletal muscle by regulating gastric inhibitory polypeptide and glucagon-like peptide 1 to improve insulin sensitivity (Miki et al., 1998). Thus, we infer that increased glucose transport in the skeletal muscle might account for the enhancement of insulin sensitivity of *SUR1*^*−/−*^ rats in spite of deficiency of insulin secretion. These inferences are also supported by previous studies that confirmed an enhanced glucose uptake in muscle when genetically deleting *Kir6.2* or *SUR2A (Chutkow et al.,* 2001*;* *Miki et al.,* 2002*)*.

Recently, there have been investigations that constructed *SUR1*^*−/−*^ mice models to clarify the mechanisms of secretion and the action of insulin (Gier et al., 2009; Szollosi et al., 2007). Belinda G, et al. elucidated that the loss of KATP channel activity may protect against streptozotocin-induced diabetes in vivo (Gier et al., 2009). Another study established a mouse model that lacked KATP channels to investigate the regulation of insulin secretion and further explored the pathology of CHI (Szollosi et al., 2007). Actually, rats are closer to human in cognitive behaviour and often serves as an ideal animal model of diabetes mellitus (Reed et al., 2000; Chis et al., 2009) and neurological disorders (McGonigle, 2014) compared with mice. Insulin secretory responses of rats are also closer to human than mice (Ma et al., 1995; Zawalich et al., 1995). SUR1 upregulation was observed in the post-injury hippocampus of rat (McGonigle, 2014), the mechanism of which will be more clearly elucidated by applying *SUR1*^*−/−*^ rat model. In addition, Reed et al. mentioned that fat-fed/STZ rat model was an ideal model for insulin resistance (Reed et al., 2000). SUR1 is primarily responsible for insulin secretion and has potential influence in insulin resistance. *SUR1*^*−/−*^ rat will contribute to clarify the specific function of SUR1 on insulin resistance. Therefore, the *SUR1*^*−/−*^ rat model might have widespread applications in different fields of study. More importantly, the characteristics of the glucose metabolism of rats upon *SUR1* gene knockout were illuminated for the first time and were completely distinguished from previous studies. Furthermore, it has been sporadically reported that apart from promoting the secretion of insulin by binding with SUR1 of KATP channel, other hypoglycaemic functions of sulfonylureas were also observed that constitute so-called extrapancreatic hypoglycaemic effects (Lee & Kim, 2007; Nordlie et al., 1999; Wu et al., 2005; Pattaranit et al., 2008). By knocking out the *SUR1* gene, a more convincing animal model was created, which will provide in vivo evidence to verify the existence of the extrapancreatic hypoglycaemic effects of sulfonylureas for future experiments. In conclusion, our *SUR1*^−/−^ rat model is a powerful tool that can be used to study the function of *SUR1* in different fields.

## Conclusion

In conclusion, the *SUR1*^*−/−*^ rat model was established for the first time in our study, and its characteristics of glucose metabolism were elucidated. Although *SUR1*^*−/−*^ rats showed glucose intolerance for KATP channel dysfunction to secret sufficient insulin, enhanced peripheral insulin responsiveness can provide a direct account for survival and protection from hyperglycaemia. Nevertheless, our study regarding the effects of *SUR1* on glucose metabolism is still part of the exploration stage, and we consider it necessary to carry out further experimental research. These new findings shed light on the glucose metabolism of *SUR1*^*−/−*^ rats, which revealed an important role of the *SUR1* gene in the endocrinological, physiological and pathological processes, with impaired insulin secretory function.

## Additional files

Additional file 1: TALEN vector for *Abcc8* deletion. The TALEN vector for *Abcc8* deletion was successfully constructed. (TIF 1889 kb)

Additional file 2: Insulin measurements of *SUR1*^*−/−*^ and wild-type rats under glucose challenge. *SUR1*^*−/−*^ rats failed to release insulin in response to glucose challenge compared with wild-type animals. (TIF 158 kb)

Additional file 3: HOMA-β level of *SUR1*^*−/−*^ and wild-type rats. HOMA-β level was significantly higher in *SUR1*^*−/−*^ rats than that of wild-type rats, which indicated an enhanced β-cell function in *SUR1*^*−/−*^ rats under non-stimulated conditions. The values denote the means ± SEM. *: *P* < 0.05. (TIF 615 kb)

## Abbreviations

^*−/−*^: Knockout
ABC: ATP-binding cassette
AUC: Area under the curve
CHI: Congenital hyperinsulinism
CNS: Central nervous system
FPG: Fasting plasma glucose
GLUT4: Glucose transporter 4
GSK3: Glycogen synthase kinase 3
HGP: Hepatic glucose production
HPF: High-power field
IOD: Integrated optical density
IPGTT: Intraperitoneal glucose tolerance test
IPITT: Intraperitoneal insulin tolerance test
KATP: ATP-sensitive K^+^
PAS: Periodic acid-Schiff
PBS: Phosphate-buffered saline
PGSK3: Phosphorylated glycogen synthase kinase 3
PHHI: Persistent hyperinsulinaemic hypoglycaemia of infancy
SEM: Standard error of mean
SUR1: Sulfonylurea receptor 1
TALEN: Transcription activator-like effector nuclease
TNF-α: Tumour necrosis factor-α
