# Supplementary figures and images for: aThe characteristics of glucose metabolism in the sulfonylurea receptor 1 knockout rat model
Source: Mol Med. 2019 Jan 7;25:2. doi: 10.1186/s10020-018-0067-9 (PMC6322298; doi:10.1186/s10020-018-0067-9)

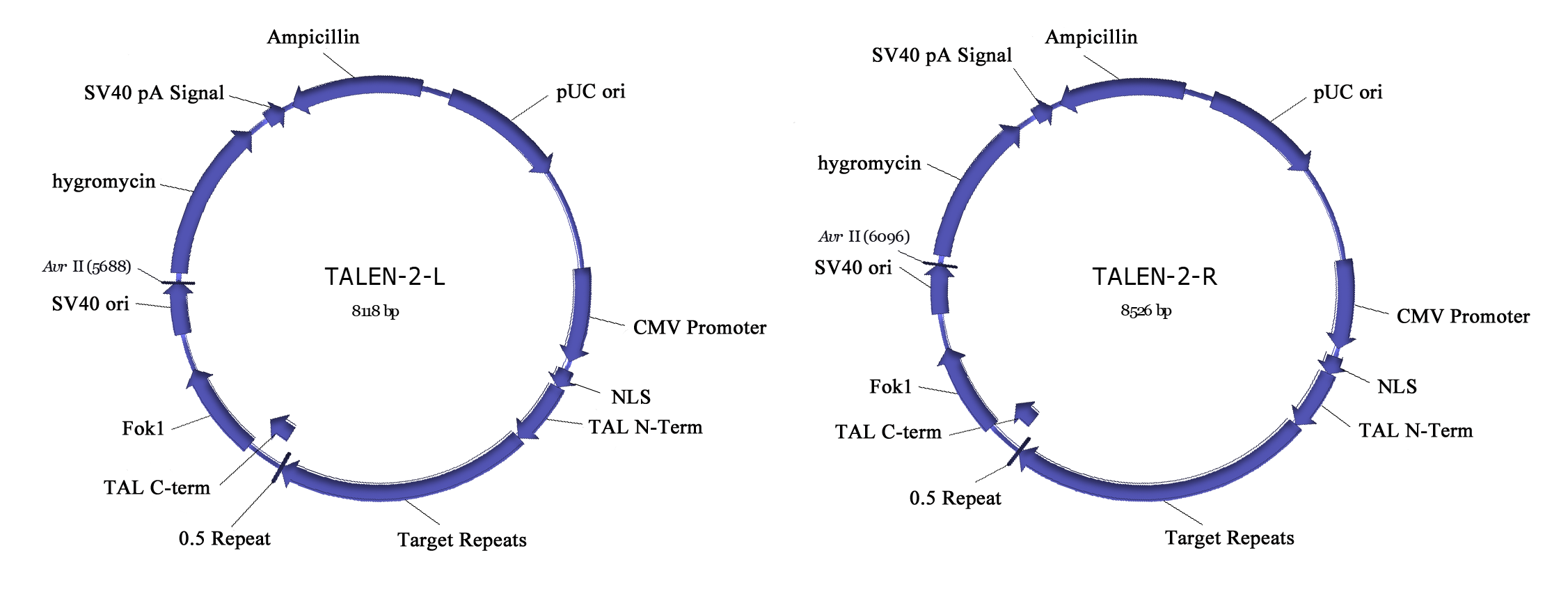

Supplement: Supplementary file 1 — TALEN vector for Abcc8 deletion. The TALEN vector for Abcc8 deletion was successfully constructed. (TIF 1889 kb) [file 10020_2018_67_MOESM1_ESM.tif]

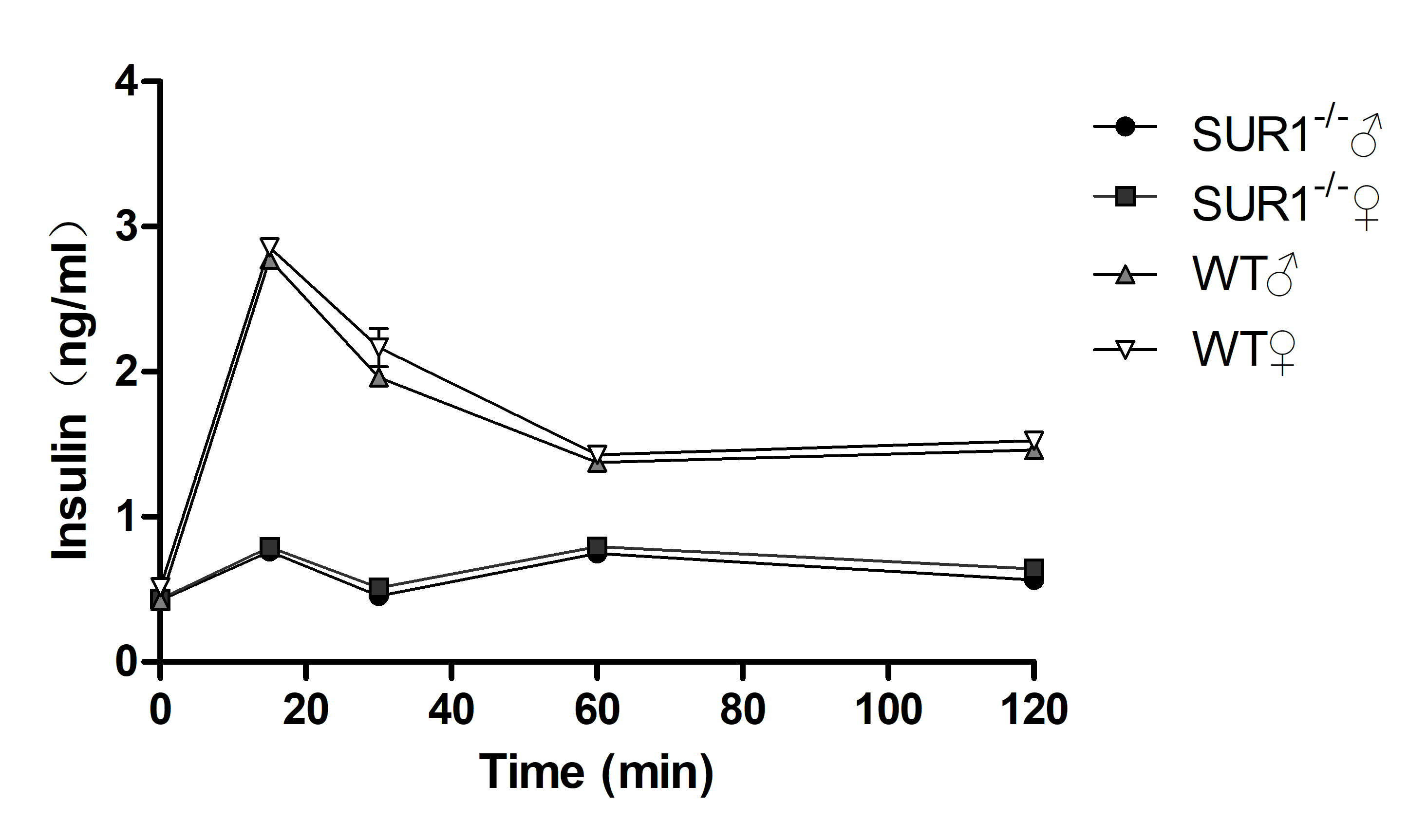

Supplement: Supplementary file 2 — Insulin measurements of SUR1−/− and wild-type rats under glucose challenge. SUR1−/− rats failed to release insulin in response to glucose challenge compared with wild-type animals. (TIF 158 kb) [file 10020_2018_67_MOESM2_ESM.tif]

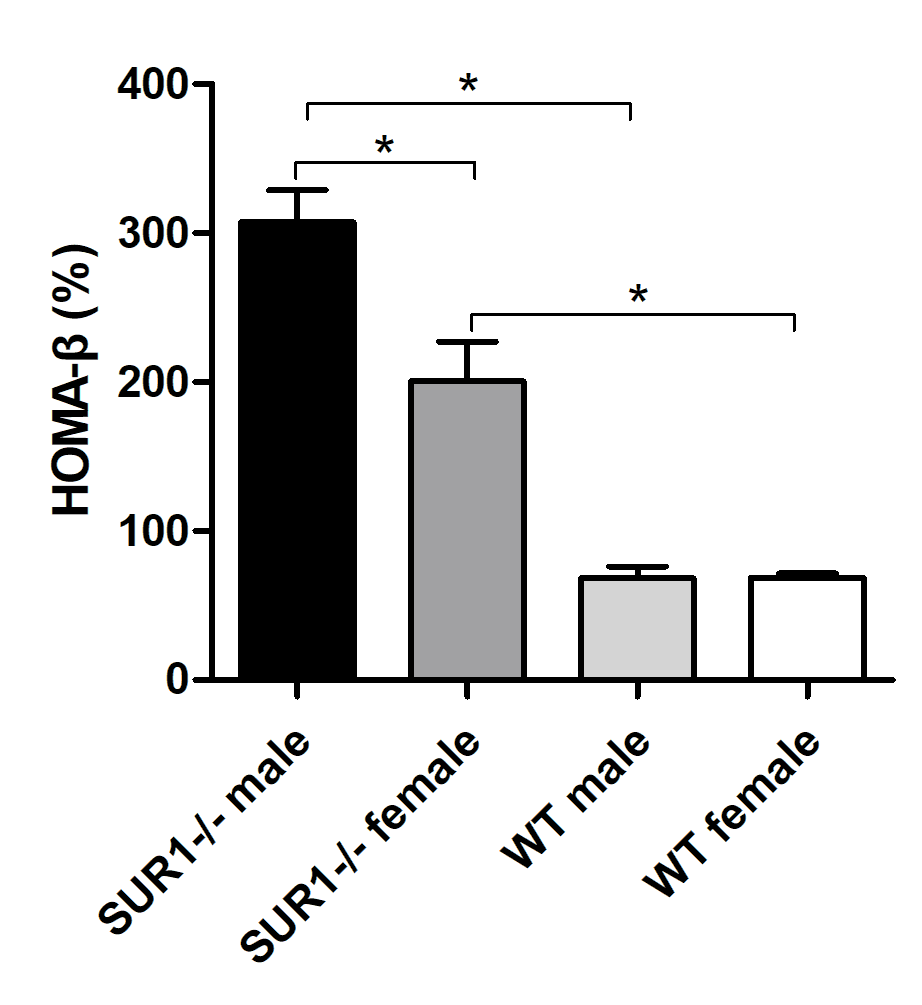

Supplement: Supplementary file 3 — HOMA-β level of SUR1−/− and wild-type rats. HOMA-β level was significantly higher in SUR1−/− rats than that of wild-type rats, which indicated an enhanced β-cell function in SUR1−/− rats under non-stimulated conditions. The values denote the means ± SEM. *: P < 0.05. (TIF 615 kb) [file 10020_2018_67_MOESM3_ESM.tif]
